# Higher serum uric acid as a risk factor for frailty in older adults: A nationwide population‐based study

**DOI:** 10.1002/jcsm.13561

**Published:** 2024-08-18

**Authors:** Min‐gu Kang, Ji Yeon Baek, Yunju Jo, Dongryeol Ryu, Il‐Young Jang, Hee‐Won Jung, Beom‐Jun Kim

**Affiliations:** ^1^ Department of Internal Medicine Chonnam National University Bitgoeul Hospital Gwangju South Korea; ^2^ Department of Internal Medicine, Division of Geriatrics, Asan Medical Center University of Ulsan College of Medicine Seoul South Korea; ^3^ Department of Biomedical Science and Engineering Gwangju Institute of Science and Technology Gwangju South Korea; ^4^ Department of Internal Medicine, Division of Endocrinology and Metabolism, Asan Medical Center University of Ulsan College of Medicine Seoul South Korea

**Keywords:** Frailty index, Oxidative stress, Pro‐aging factor, Systemic inflammation, Uric acid

## Abstract

**Background:**

Uric acid (UA), the terminal breakdown product of purine metabolism, possesses contradictory roles, functioning both as an inflammatory mediator and as an antioxidant. Its clinical relevance, particularly in geriatric populations, remains a topic of ongoing debate. Aiming to elucidate whether circulating UA is detrimental or beneficial to human health, we investigate the association between serum UA concentrations and the frailty index—a comprehensive measure of biological aging in a nationally representative cohort of community‐dwelling older adults.

**Methods:**

We conducted a population‐based, cross‐sectional study utilizing data from the Korea National Health and Nutrition Examination Survey. The sample included 4268 participants aged 65 years and above. A deficit accumulation frailty index (FI) was constructed using 38 items that assess physical, cognitive, psychological, and social domains. Based on the FI, participants were categorized into non‐frail (FI ≤ 0.15), pre‐frail (0.15 < FI ≤ 0.25), or frail (FI > 0.25). Serum UA levels were quantified through a colorimetric enzymatic assay.

**Results:**

After controlling for confounders such as age, sex, socioeconomic status (including income and education level), lifestyle factors (smoking status), and medical history (hypertension, diabetes, dyslipidemia, stroke, cardiovascular diseases), and body mass index, serum UA levels were observed to be significantly higher in frail participants compared with their non‐frail counterparts (*P* < 0.001). Furthermore, serum UA concentrations demonstrated a positive correlation with the FI (*P* < 0.001), and the odds ratio for frailty per 1 mg/dL increase in serum UA was 1.22 (*P* < 0.001). Additionally, older adults in the highest quartile of UA levels exhibited a significantly higher FI and 1.66‐fold increased odds of frailty compared with those in the lowest quartile (*P* = 0.011 and *P* = 0.005, respectively).

**Conclusions:**

These findings suggest that elevated circulating UA levels may act as a pro‐aging factor rather than an anti‐aging one in older adults, highlighting its potential role in accelerating biological aging. The data further support the utility of serum UA as a potential blood‐based biomarker for frailty in this demographic, contributing to the expanding evidence on its significance in geriatric health assessments.

## Introduction

Frailty in older adults is a critical geriatric syndrome characterized by diminished physiological capacity, leading to increased vulnerability to various stressors, and is more reflective of an individual's holistic well‐being and functional prowess than merely chronological age.^1^, ^2^ Its clinical importance is highlighted by its association with adverse health outcomes such as falls, disability, and death, necessitating robust assessment tools.^2^, ^3^ Although various tools, such as the Edmonton Frail Scale, Tilburg Frailty Indicator, and Clinical Frailty Scale, have been proposed for assessing frailty,^4^, ^5^ the ‘phenotypic frailty’ or Fried criteria focuses predominantly on physical aspects and is widely used due to its simplicity.^6^ However, the ‘frailty index’ developed by Rockwood et al.,^7^, ^8^ which includes a broader array of deficits such as cognitive, psychological, and social factors, is recognized as a more comprehensive and superior predictor of critical outcomes like hospitalization and mortality.^9^, ^10^ In fact, a recent longitudinal study has underscored the frailty index as the most reliable indicator of biological age,^11^ promoting its use as a primary endpoint in clinical research on aging. This approach not only enhances the accuracy of frailty assessments but also supports more effective interventions to improve quality of life and extend healthy living among the older adults, highlighting the need for continuous advancements in frailty research, particularly through the development and integration of reliable biomarkers.

Experimental investigations have revealed that uric acid (UA), a by‐product of purine metabolism, can induce inflammation, oxidative stress, vasoconstriction, and endothelial dysfunction.^12^ Moreover, epidemiological research has consistently demonstrated associations between elevated serum UA levels and an increased risk of diabetes, hypertension, cardiovascular disorders, metabolic syndrome, and kidney disease.^13^, ^14^, ^15^ As such, UA is traditionally considered a risk factor for a diverse array of diseases, predominantly those related to aging. Contrarily, there is growing evidence to suggest a potentially beneficial physiological role for UA as an antioxidant, which might contribute to its free radical scavenging capabilities, thereby possibly extending life expectancy.^16^, ^17^, ^18^ Indeed, higher concentrations of serum UA have been linked with slower progression of neurodegenerative conditions such as Huntington's disease, Parkinson's disease, and mild cognitive impairment.^19^, ^20^ Given these dual implications—both potentially beneficial and detrimental—of serum UA on human health, its role in the aging process remains ambiguous. To address these unresolved questions, our study investigates the relationship between circulating UA concentrations and the frailty index, a robust indicator of biological aging, in a comprehensive, nationally representative cohort including community‐dwelling older adults.

## Methods

### Study population

This cross‐sectional analysis utilized data from the Korea National Health and Nutrition Examination Survey (KNHANES), conducted between 2016 and 2018. KNHANES, initiated in 1998, aims to assess the health and nutritional status of the Korean population nationwide. It monitors trends in health risk factors, the prevalence of significant chronic diseases, and serves as a foundation for developing and evaluating health policies and programs in Korea.^21^ KNHANES employs a complex, multistage probability sampling design to represent the entire non‐institutionalized civilian population. Annually, the survey selects primary sample units (PSUs) from census blocks or resident registration addresses, typically comprising 50 to 60 households. From each PSU, 20–25 households are chosen for participation through field surveys. All individuals aged 1 year and above residing in these households are included in the survey.

**Figure 1:**
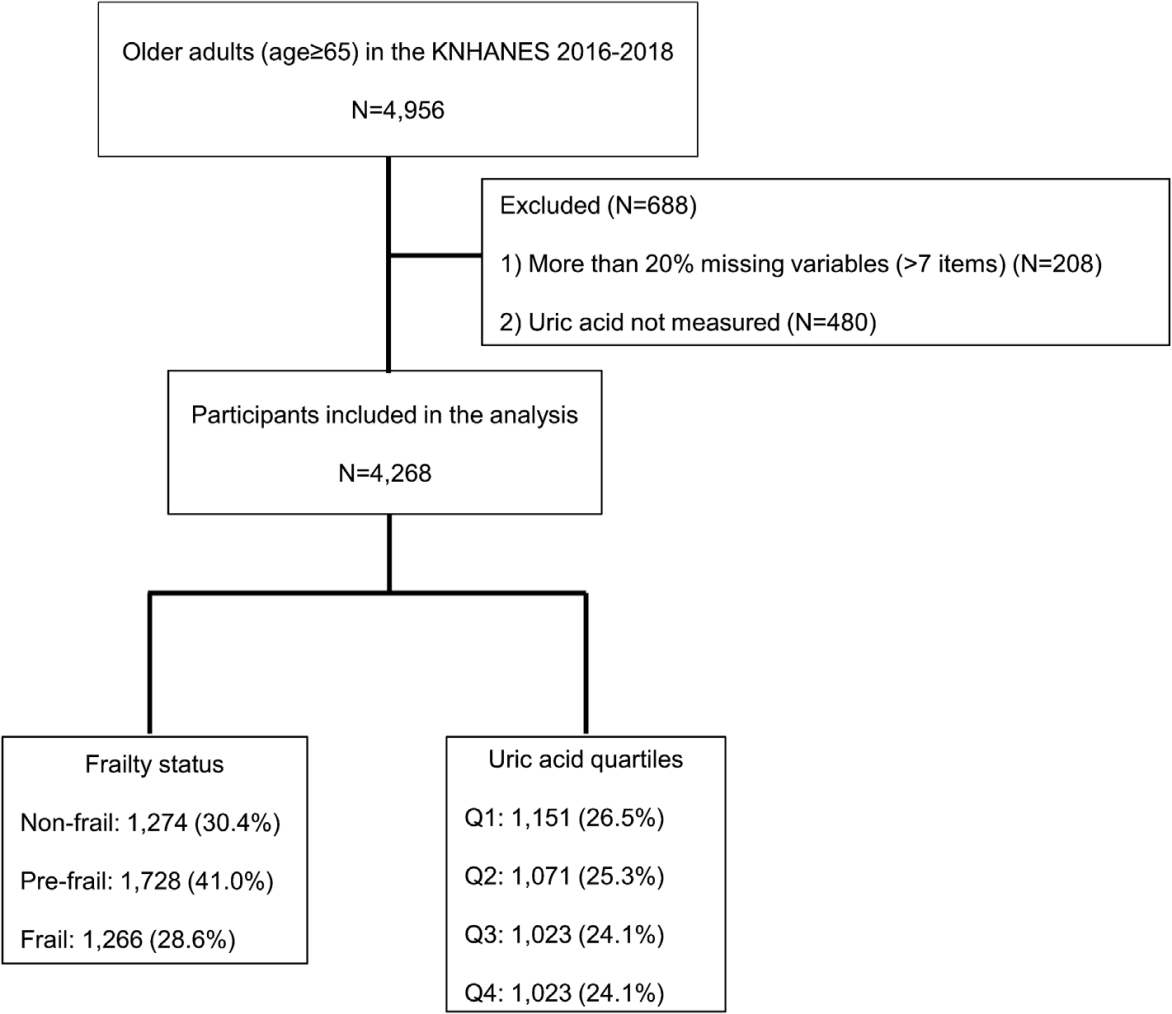
Flow diagram of the study participants. Q, quartile. Uric acid quartiles: Q1 = serum uric acid ≤ 4.1 (mg/dL), Q2 = 4.1 < serum uric acid ≤ 4.9, Q3 = 4.9 < serum uric acid ≤ 5.8, Q4 = serum uric acid > 5.8.

During the study period, 4956 older adults (aged ≥65 years) participated in KNHANES. After excluding 208 individuals missing over 20% (more than seven items) of the frailty assessment data and 480 participants without UA measurements, 4268 participants were included in the analysis (Figure 1). Our study did not exclude individuals with specific conditions such as gout or kidney stones to ensure the generalizability of our results and to align with the objective of using a comprehensive frailty index to capture the true burden of health deficits in older adults. All participants had provided written informed consent. Personal data collected were de‐identified prior to public release. The study received approval from the Institutional Review Board of Chonnam National University Bitgoeul Hospital, with a waiver for additional informed consent (IRB No. CNUBH‐2024‐005), adhering to the Declaration of Helsinki.

### Serum uric acid concentration measurement

Blood samples for serum UA level determinations were collected from participants aged 10 years and older, with informed consent. After fasting overnight for at least 8 h, blood was drawn from the antecubital vein in the morning. Samples were immediately refrigerated, transported to the Central Testing Institute (Neodin Medical, Inc., Seoul, Korea), and analysed within 24 h. Serum UA concentrations were measured using a colorimetric enzymatic method (uricase) with the Hitachi Automatic Analyser 7600‐210 (Hitachi, Japan). The analytical kit used had a detection limit of less than 1.0 mg/dL and a coefficient of variation (CV) under 5%. The quality control details are publicly available on the KNHANES homepage (https://knhanes.kdca.go.kr).

### Frailty‐related factors evaluation

Blood pressure was measured on the right arm using a Baumanometer® Wall Unit 33(0850) (W.A. Baum, Copiague, NY, USA) by trained nurses. Participants were instructed to sit quietly for at least 5 min before measurements were taken. Blood pressure was recorded three times, with the final systolic and diastolic values calculated as the average of the second and third readings. Blood samples were collected as part of the survey. Body mass index (BMI) was determined by dividing the body weight in kilograms (kg) by the square of the height in meters (m^2^). Socioeconomic and lifestyle data were collected via self‐reported questionnaires. Household income was categorized into quartiles: the lowest quartile for households earning <$680 per month; the lower‐middle quartile for those earning between $680 and $1360; the upper‐middle quartile for incomes between $1360 and $2230; and the highest quartile for incomes above $2230 per month. Education levels were divided into four categories: elementary or lower, middle school, high school, and college or higher. Smoking status was assessed by identifying individuals who had smoked at least five packs of cigarettes in their lifetime and were current smokers. Participants were also classified by medical conditions based on diagnoses received from a physician.

### Frailty index

The frailty index in our study was developed following a standardized methodology, as outlined in previous literature,^22^ and was adapted from existing frailty indices constructed using data from the KNHANES.^23^, ^24^ This index quantifies frailty on a continuous scale from 0 (indicating optimal health) to 1 (indicating severe frailty).^10^

From 2016 to 2018, the KNHANES included 38 items in the frailty index, encompassing a wide spectrum of factors such as co‐morbidities, functional abilities, signs and symptoms, and laboratory results. The co‐morbidities included in the index are anaemia, arthritis, bronchial asthma, cancer, cardiovascular disease, cataract, chronic obstructive pulmonary disease, depression, diabetes, dyslipidaemia, hypertension, and stroke. Functional abilities were evaluated based on criteria such as physical inactivity, reduced exercise capacity, limitations in activities of daily living, social activity restrictions, inability to self‐care, hearing impairment, and difficulty in chewing. Signs and symptoms assessed included pain or discomfort, weight loss, depression or anxiety, suicidal thoughts, and stress. Laboratory measures incorporated into the index included systolic and diastolic blood pressure, heart rate regularity, pulmonary function, haemoglobin levels, blood urea nitrogen, creatinine, total cholesterol, triglycerides, high‐density lipoprotein cholesterol, fasting glucose, and urine protein levels. Additionally, current smoking status and BMI were included (Table S1). Participants were stratified into three categories based on the frailty index values: non‐frail (frailty index ≤ 0.15), pre‐frail (0.15 < frailty index ≤ 0.25), and frail (frailty index > 0.25), consistent with criteria from prior studies.^25^, ^26^

### Statistical analysis

This study utilized complex sample analysis methods with assigned weights to derive national‐level statistical estimates. A pooled analysis of the annual surveys was conducted, treating each year's sample as independent. Data were reported as means with standard errors (SEs) or counts with percentages, unless specified otherwise. Baseline characteristics of the study participants were analysed using a general linear model for continuous variables and cross‐tabulation for categorical variables. Confounding variables selected due to their clinical relevance and statistical significance in univariate analyses included age, sex, income, education level, current smoking status, hypertension, diabetes, dyslipidaemia, stroke, cardiovascular diseases, and BMI. Linear regression analysis was employed to examine the association between higher serum UA levels and an increased frailty index, with the frailty index serving as the dependent variable and serum UA as the independent variable. The risk of pre‐frailty and frailty in relation to serum UA levels or serum UA quartiles was explored using multiple logistic regression. Additionally, differences in the frailty index across serum UA quartiles were assessed with a general linear model. All analyses were two‐tailed, with statistical significance set at *P* < 0.05, and performed using SPSS version 21.0 (IBM Corporation, Armonk, NY, USA).

## Results

### Baseline characteristics of study participants

Table 1 presents the baseline characteristics of 4268 participants aged 65 years and older. Of these, 1274 (30.4%) were categorized as non‐frail, 1728 (41.0%) as pre‐frail, and 1266 (28.6%) as frail. The distribution of gender within these groups was significantly different: 666 (51.8%) non‐frail, 752 (43.0%) pre‐frail, and 450 (33.9%) frail participants were men (*P* < 0.001). The mean ages were 71.0 years for non‐frail, 72.9 years for pre‐frail, and 74.4 years for frail individuals, showing a statistically significant increase with greater frailty (*P* < 0.001). Socioeconomic and health characteristics worsened progressively from non‐frail to frail groups, including lower income, less education, higher numbers of smokers, and a higher prevalence of hypertension, diabetes, dyslipidaemia, stroke, cardiovascular diseases, and increased BMI (all *P* < 0.001).

**Table 1:** Baseline characteristics of the study participants according to frailty status

	Non‐frail (*N* = 1274)	Pre‐frail (*N* = 1728)	Frail (*N* = 1266)	*P* value
Age (years), mean (SE)	**71.0 (0.2)**	**72.9 (0.1)**	**74.4 (0.2)**	**<0.001**
Sex (male sex), *n* (%)	**666 (51.8%)**	**752 (43.0%)**	**450 (33.9%)**	**<0.001**
Income quartile (low/mid‐low/mid‐high/high), %	**36.6%/30.6%/17.6%/15.2%**	**44.6%/26.4%/17.6%/11.4%**	**61.3%/22.1%/10.6%/5.9%**	**<0.001**
Level of education (1st/2nd/3rd/4th), %	**44.6%/18.2%/20.8%/16.5%**	**57.3%/14.6%/17.7%/10.4%**	**71.2%/12.7%/11.6%/4.5%**	**<0.001**
Smoking, *n* (%)	**67 (5.5%)**	**174 (10.4%)**	**153 (11.7%)**	**<0.001**
Hypertension, *n* (%)	**581 (45.2%)**	**1134 (65.1%)**	**994 (80.8%)**	**<0.001**
Diabetes, *n* (%)	**130 (10.2%)**	**454 (27.0%)**	**496 (41.4%)**	**<0.001**
Dyslipidaemia, *n* (%)	**277 (22.3%)**	**594 (35.7%)**	**540 (43.9%)**	**<0.001**
Stroke, *n* (%)	**22 (1.8%)**	**77 (5.0%)**	**155 (12.3%)**	**<0.001**
Cardiovascular disease (MI, angina), *n* (%)	**39 (2.6%)**	**122 (6.8%)**	**188 (14.1%)**	**<0.001**
BMI (kg/m^2^), mean (SE)	**23.5 (0.1)**	**24.2 (0.1)**	**24.9 (0.1)**	**<0.001**

Continuous and categorical variables were compared using general linear model and crosstabs analyses in a complex sample analysis method, respectively. Bold numbers indicate statistically significant values.

BMI, body mass index; MI, myocardial infarction; SE, standard error.

### Serum uric acid concentrations and frailty status

**Figure 2:**
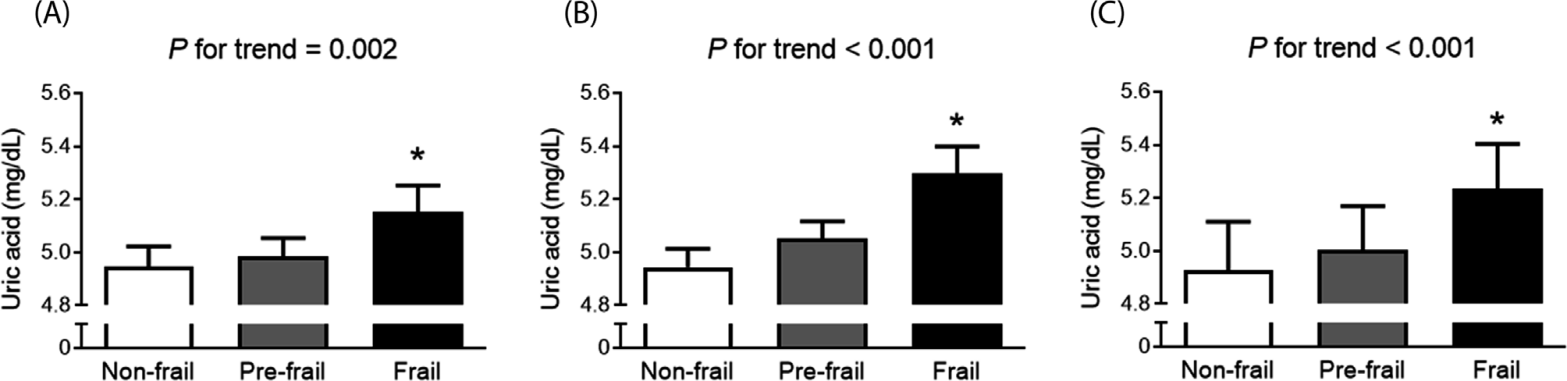
Differences in serum uric acid levels according to the frailty status. (A) Unadjusted, (B) age and sex adjusted, and (C) multivariable (age, sex, income, level of education, smoking, hypertension, diabetes, dyslipidaemia, stroke, cardiovascular diseases, and body mass index) adjusted. The estimated means with 95% confidence intervals were generated and compared using general linear model analysis in a complex sample analysis method. Asterisk indicates statistically significant difference from the non‐frail group.

Differences in serum UA concentrations by frailty status were analysed using a general linear model within a complex sample analysis framework (Figure 2). Prior to adjustment, a linear increase in UA levels was observed as frailty severity increased from non‐frail to frail (*P* for trend = 0.002), with frail adults showing significantly higher serum UA levels compared with non‐frail counterparts (*P* = 0.002). These differences persisted after adjustments for age, sex, and further adjustments for socioeconomic and health factors including income, education, smoking status, hypertension, diabetes, dyslipidaemia, stroke, cardiovascular diseases, and BMI (both *P* < 0.001).

### Association between serum uric acid levels and frailty index

Linear regression analyses indicated that higher serum UA levels were consistently associated with an elevated frailty index, both before and after adjusting for potential confounders (Table 2; *P* < 0.001 to 0.001).

**Table 2:** Multiple linear regression analysis to determine whether serum uric acid level is independently associated with frailty index

Adjustment	Dependent variable: frailty index		
	β	SE	*P* value
Unadjusted	**0.004**	**0.001**	**0.001**
Age and sex	**0.008**	**0.001**	**<0.001**
Multivariable	**0.005**	**0.001**	**<0.001**

General linear model analysis was performed with frailty index as a dependent variable, and with serum uric acid level (mg/dL) as an independent variable. Multivariable adjustment model includes age, sex, income, level of education, smoking, hypertension, diabetes, dyslipidaemia, stroke, cardiovascular diseases, and body mass index as confounding factors. Bold numbers indicate statistically significant values.

β, regression coefficient; SE, standard error.

### Risk of frailty in relation to serum uric acid levels

Multiple logistic regression analyses explored the risk of frailty relative to serum UA levels (Table 3). An increase of 1 mg/dL in serum UA was associated with a non‐significant increase in the odds of pre‐frailty (*P* = 0.061 to 0.499), except in the age‐ and sex‐adjusted model (*P* = 0.020). However, the same increase was associated with a significant risk of becoming frail, with crude odds ratios of 1.12 (*P* = 0.001), and a 25% and 22% increase in risk in the age‐ and sex‐adjusted and multivariable adjusted models, respectively (both *P* < 0.001).

**Table 3:** Logistic regression analyses to determine the odds ratios for pre‐frail and frail status according to serum uric acid level

Adjustment	Pre‐frail	*P* value	Frail	*P* value
	Odds ratio (95% CIs)^a^		Odds ratio (95% CIs)^a^	
Unadjusted	1.02 (0.96–1.09)	0.499	**1.12 (1.05–1.20)**	**0.001**
Age and sex	**1.08 (1.01–1.15)**	**0.020**	**1.25 (1.16–1.35)**	**<0.001**
Multivariable	1.07 (1.00–1.15)	0.061	**1.22 (1.12–1.33)**	**<0.001**

Multivariable adjustment model includes age, sex, income, level of education, smoking, hypertension, diabetes, dyslipidaemia, stroke, cardiovascular diseases, and body mass index as confounding factors. Bold numbers indicate statistically significant values.

CI, confidence interval.

^a^ Per 1 mg/dL increment in serum uric acid level.

### Threshold effect of serum uric acid on frailty

**Figure 3:**
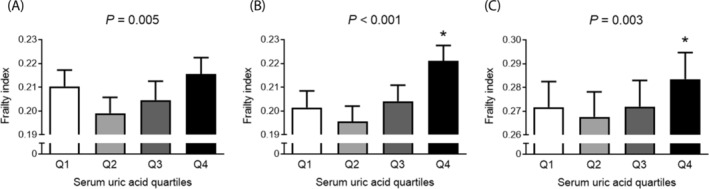
Differences in frailty index according to serum uric acid quartiles. (A) Unadjusted, (B) age and sex adjusted, and (C) multivariable (age, sex, income, level of education, smoking, hypertension, diabetes, dyslipidaemia, stroke, cardiovascular diseases, and body mass index) adjusted. The estimated means with 95% confidence intervals were generated and compared using general linear model analysis in a complex sample analysis method. Asterisk indicates statistically significant difference from the Q1 (lowest quartile). Q, quartile. Uric acid quartiles: Q1 = serum uric acid ≤ 4.1 (mg/dL), Q2 = 4.1 < serum uric acid ≤ 4.9, Q3 = 4.9 < serum uric acid ≤ 5.8, Q4 = serum uric acid > 5.8.

**Figure 4:**
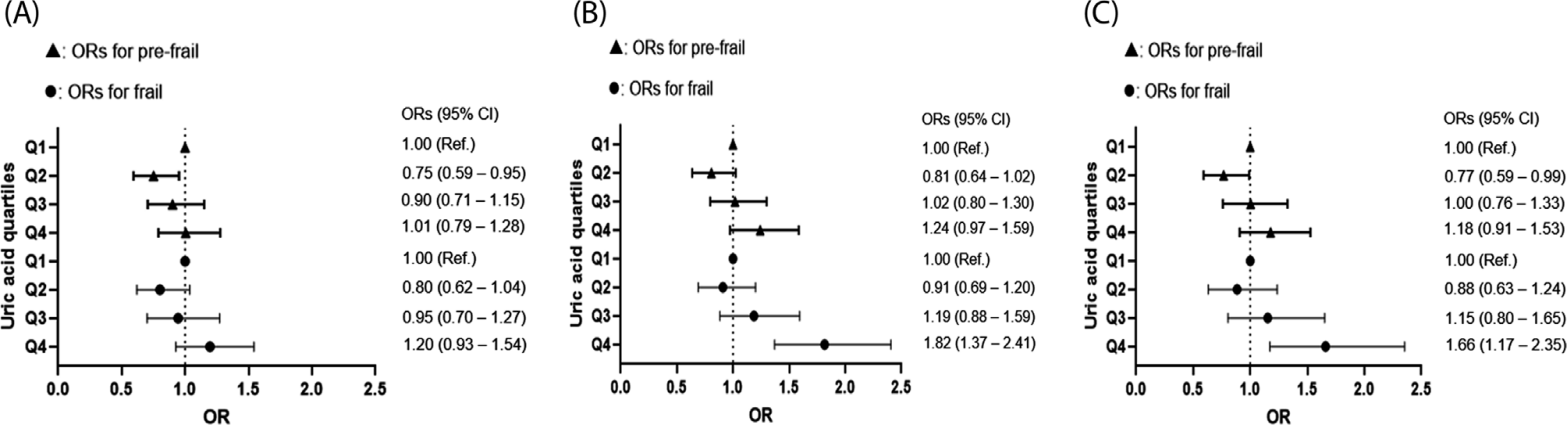
Logistic regression analyses to determine the odds ratios for pre‐frail and frail status according to serum uric acid quartiles. (A) Unadjusted, (B) age and sex adjusted, and (C) multivariable (age, sex, income quartile, level of education, smoking, hypertension, diabetes, dyslipidaemia, stroke, cardiovascular diseases, and body mass index) adjusted. OR, odds ratio; CI, confidence interval; Q, quartile. Uric acid quartiles: Q1 = serum uric acid ≤ 4.1 (mg/dL), Q2 = 4.1 < serum uric acid ≤ 4.9, Q3 = 4.9 < serum uric acid ≤ 5.8, Q4 = serum uric acid > 5.8.

To investigate potential threshold effects, participants were divided into quartiles based on serum UA levels (Figure 3). The frailty index displayed a J‐shaped curve across these quartiles. Older adults in the highest quartile (Q4, serum UA > 5.8 mg/dL) exhibited a significantly higher frailty index than those in the lowest quartile (Q1, serum UA ≤ 4.1 mg/dL) in both the age‐ and sex‐adjusted and multivariable adjusted models (*P* < 0.001 and *P* = 0.011, respectively). Furthermore, logistic regression analyses revealed that participants in Q4 had a 1.82‐fold higher odds ratio for frailty compared with those in Q1 in the age‐ and sex‐adjusted model (Figure 4; *P* < 0.001), and this elevated risk remained significant even after adjusting for all potential confounders (*P* = 0.005).

### Risk of frailty according to the presence of hyperuricaemia

The widely accepted upper limit cut‐off values for UA are 7 mg/dL for men and 6 mg/dL for women, with hyperuricaemia defined as levels exceeding these thresholds. When participants were divided into two groups based on UA levels, separately for men and women, we found that participants in the hyperuricaemia group exhibited a significantly higher frailty index compared with those in the reference group, even after adjusting for confounding factors, in both men and women (Figure S1; all *P* < 0.001). Furthermore, logistic regression analyses of the unadjusted model revealed that older adults in the hyperuricaemia group had 2.33‐fold and 4.30‐fold higher odds ratios for frailty in men and women, respectively, compared with those in the reference group (Figure S2; both *P* < 0.001). These elevated odds ratios for frailty in the hyperuricaemia group remained statistically significant even after adjusting for potential confounders such as age, income, education level, smoking status, hypertension, diabetes, dyslipidaemia, stroke, cardiovascular diseases, and BMI in both men and women (*P* = 0.016 and *P* < 0.001, respectively).

## Discussion

UA, known for its dual role in the inflammatory response and as an antioxidant, presents a paradox in its clinical implications, particularly in geriatric populations where its significance remains debated. This study, involving community‐dwelling older adults, demonstrated that serum UA levels were significantly higher in frail participants compared with their non‐frail counterparts. Furthermore, the data reveal a significant correlation between increased serum UA levels and both an elevated frailty index and a higher risk of developing frailty. These results clinically underscore the potential of circulating UA as a pro‐aging factor rather than an anti‐aging one in this demographic. Moreover, this research adds to the growing body of evidence that suggests serum UA could serve as a potential blood‐based biomarker for detecting frailty in older adults.

While both the phenotype model and the cumulative deficit model have demonstrated reliability in predicting the natural progression of diseases and responses to interventions,^2^ the Rockwood frailty index offers distinct advantages. It encompasses a more extensive array of health deficits, providing a comprehensive assessment of frailty.^7^, ^8^ This comprehensive approach affords a holistic perspective on an individual's health status, in contrast to the phenotypic model, which is limited to five physical criteria and categorizes frailty in a binary manner.^6^ The continuous nature of the frailty index allows for a nuanced representation of the spectrum of frailty, facilitating early detection of incremental health deteriorations. Such sensitivity enhances its utility in forecasting critical health outcomes, including hospitalizations and mortality rates.^8^, ^9^, ^10^ Importantly, the KNHANES provides a robust dataset that is crucial for constructing the Rockwood frailty index, capturing diverse factors such as co‐morbidities, cognitive functions, and psychosocial elements.^21^ Given its comprehensive and representative data, KNHANES serves as an ideal large‐scale, nationwide cohort for clinical research on frailty, a field of growing importance in the context of an aging population.

Frailty, characterized by muscular weakness, diminished energy, reduced physical activity, and decreased resilience, is increasingly recognized as clinically significant in societies experiencing profound aging.^1^ Consequently, there is an escalating interest in identifying potential biomarkers for early detection of individuals at high risk of developing frailty.^27^ Despite numerous clinical investigations into the association between circulating UA levels and various chronic diseases that may predispose individuals to frailty, research directly analysing its impact on frailty *per se* remains sparse. To date, only a single study has reported a correlation, indicating an increased risk of frailty concurrent with rising serum UA concentrations in older adults.^28^ This study, however, primarily employs the criteria defined by Fried et al.,^6^ focusing largely on physical dimensions and potentially insufficient for establishing a comprehensive correlation between frailty—which encompasses overall well‐being and functional capacity—and UA levels. Our study addresses this gap by being the first to utilize robust national datasets in human UA research, constructing a frailty index that incorporates the cumulative impact of medical, functional, and psychosocial deficits associated with aging. This approach provides compelling clinical evidence supporting the role of UA as a pro‐aging factor in the development of frailty.

The association between high serum UA concentrations and increased risk of frailty can be attributed to several underlying mechanisms. Most importantly, as mentioned earlier, UA can induce systemic inflammation and oxidative stress by promoting the formation of reactive oxygen species (ROS) and activating inflammatory pathways, leading to cellular damage and physiological decline, which are central to frailty.^12^, ^16^ Second, UA is associated with endothelial dysfunction, which impairs blood flow and nutrient delivery, exacerbating muscle weakness and physical decline.^29^ Third, elevated UA levels also correlate with insulin resistance, contributing to muscle protein breakdown and reduced physical capability.^30^ Finally, high UA levels can adversely affect renal function, impacting the body's ability to eliminate waste and maintain fluid balance, thereby enhancing fatigue and decreasing physical activity, which are critical factors in the development of frailty.^31^, ^32^ Collectively, these pathways underscore the multifaceted role of UA in promoting age‐related physiological challenges and advancing the frailty syndrome.

A particularly intriguing finding in our study was the J‐shaped curve observed in the changes of the frailty index across serum UA quartiles. Specifically, compared with older adults in the lowest quartile of UA, those in the second quartile exhibited a lower tendency in frailty index, which gradually increased, resulting in a statistically significant rise in the highest quartile group (Figure 3). Similarly, the risk of frailty, assessed through logistic regression analysis, showed a decreasing trend in the second quartile, followed by an increase in the third quartile, and a statistically significant elevation in the fourth quartile (Figure 4). These results align with numerous epidemiological studies demonstrating a J‐shaped association between serum UA concentrations and cardiovascular events as well as all‐cause mortality.^33^, ^34^ The findings suggest that while circulating UA at optimal levels may serve a protective antioxidant role, excessive concentrations can trigger adverse health effects through threshold mechanisms. Indeed, there is supporting evidence that UA transitions from an antioxidant to a pro‐oxidant state under hyperuricaemic conditions, influenced by factors such as the local oxidant environment, acidity, or depletion of other antioxidants.^35^ Identifying the precise serum UA concentrations that are beneficial or harmful is crucial and warrants further clinical investigation.

The present study demonstrates a positive correlation between elevated serum UA levels and an increased frailty index in older adults. However, the question remains whether high UA levels are causative, associative, or merely a compensatory response to frailty. To address this limitation, it is essential to investigate the impact of UA‐lowering interventions on frailty measures. Notably, allopurinol has shown promise in improving both physical and cognitive functional outcomes in elderly populations.^36^, ^37^, ^38^ Consequently, future research should focus on interventional trials utilizing allopurinol or other UA‐lowering agents to assess their effects on frailty measures. Such studies are crucial to determine whether lowering UA levels can alleviate frailty and improve overall functional health in older adults, thereby clarifying the potential causative role of UA in the development of frailty.

While our study primarily focuses on the association between serum UA levels and frailty *per se* in older adults, we acknowledge that higher UA levels are often linked to various dietary habits and medical conditions. Elevated UA levels can be influenced by high purine intake, fructose consumption, and obesity, as well as by conditions such as hypertension, diabetes, and kidney disease.^39^ Although these factors were not the central focus of our research, understanding their impact on UA levels is crucial. Avoiding dietary and lifestyle choices that increase UA can help mitigate frailty by reducing the risk of elevated UA levels. Therefore, public health strategies aiming to control UA levels through nutrition and disease management may play a significant role in preventing frailty in the aging population.

Our study's principal strength is the utilization of complex sample analysis methods with assigned weights, which facilitated the estimation of national‐level statistics, thereby enhancing the generalizability of our findings. Moreover, the extensive sample size allowed for adjustments for a broad range of confounding factors, increasing the statistical robustness of our results. Nonetheless, it is crucial to acknowledge certain limitations that should be considered when interpreting our data. A significant limitation of our study is its cross‐sectional design, which impedes the establishment of a causal relationship between serum UA levels and frailty. Furthermore, the KNHANES dataset does not include measurements of systemic inflammation and oxidative stress in the collected samples, limiting our ability to confirm whether the observed increase in frailty risk due to high UA concentrations is directly attributable to these factors. Another limitation is our reliance on self‐reported data, which may introduce recall and social desirability biases. In addition, while the KNHANES provides comprehensive health‐related data, it did not specifically include assessments of participants' consumption of purine‐rich foods that affect serum UA levels. Lastly, as our study exclusively involved a Korean population, the applicability of our findings to other demographic groups, particularly Caucasians, may be limited.

In conclusion, findings from a nationally representative cohort indicate that elevated serum UA levels are robustly linked with an increased frailty index that encompasses physical, cognitive, psychological, and social dimensions, as well as an elevated risk of frailty among older adults. This evidence supports the notion that high circulating UA levels may serve as a pro‐aging factor rather than providing anti‐aging benefits in this population. Future studies are warranted to explore whether interventions aimed at lowering UA levels can ameliorate oxidative stress and inflammatory responses, ultimately contributing to improved biological aging outcomes.

## Funding

This research was supported by grants from the Korea Health Technology R&D Project through the Korea Health Industry Development Institute (KHIDI), funded by the Ministry of Health & Welfare, Republic of Korea (grant number: RS‐2024‐00401934) and from the Asan Institute for Life Science, Asan Medical Center, Seoul, Republic of Korea (grant number: 2022IP0077).

## Conflict of interest

Hee‐Won Jung cofounded Dyphi Inc, a start‐up company developing sensor technologies for human movement and robotics. Min‐gu Kang, Ji Yeon Baek, Yunju Jo, Dongryeol Ryu, Il‐Young Jang, and Beom‐Jun Kim declare that they have no conflict of interest.

## Supporting information


**Table S1.** Variables included in the frailty index

**Figure S1.** Differences in frailty index according to serum uric acid status. A) unadjusted (Men), B) multivariable (age, income, level of education, smoking, hypertension, diabetes, dyslipidemia, stroke, cardiovascular diseases, and body mass index) adjusted (Men). C) unadjusted (Women), D) multivariable (age, income, level of education, smoking, hypertension, diabetes, dyslipidemia, stroke, cardiovascular diseases, and body mass index) adjusted (Women). The estimated means with 95% confidence intervals were generated and compared using general linear model analysis in a complex sample analysis method. Asterisk indicates statistically significant difference from the reference level. Reference level (Men) = serum uric acid ≤ 7.0 mg/dL, Hyperuricemia (Men) = serum uric acid > 7.0 mg/dL, Reference level (Women) = serum uric acid ≤ 6.0 mg/dL, Hyperuricemia (Women) = serum uric acid > 6.0 mg/dL.

**Figure S2.** Logistic regression analyses to determine the odds ratios for pre‐frail and frail status according to serum uric acid levels. A) unadjusted (Men), B) multivariable (age, income, level of education, smoking, hypertension, diabetes, dyslipidemia, stroke, cardiovascular diseases, and body mass index) adjusted (Men). C) unadjusted (Women), D) multivariable (age, income, level of education, smoking, hypertension, diabetes, dyslipidemia, stroke, cardiovascular diseases, and body mass index) adjusted (Women). OR, odds ratio; CI, confidence interval. Reference level (Men) = serum uric acid ≤ 7.0 mg/dL, Hyperuricemia (Men) = serum uric acid > 7.0 mg/dL, Reference level (Women) = serum uric acid ≤ 6.0 mg/dL, Hyperuricemia (Women) = serum uric acid > 6.0 mg/dL.

## References

[1] Lee H , Lee E , Jang IY . Frailty and comprehensive geriatric assessment. J Korean Med Sci 2020;35:e16. 31950775 10.3346/jkms.2020.35.e16 PMC6970074

[2] Clegg A , Young J , Iliffe S , Rikkert MO , Rockwood K . Frailty in elderly people. Lancet 2013;381:752–762. 23395245 10.1016/S0140-6736(12)62167-9 PMC4098658

[3] Vermeiren S , Vella‐Azzopardi R , Beckwée D , Habbig AK , Scafoglieri A , Jansen B , et al. Frailty and the prediction of negative health outcomes: a meta‐analysis. J Am Med Dir Assoc 2016;17:1163.e1–1163.e17. 10.1016/j.jamda.2016.09.010 27886869

[4] Aucoin SD , Hao M , Sohi R , Shaw J , Bentov I , Walker D , et al. Accuracy and feasibility of clinically applied frailty instruments before surgery: a systematic review and meta‐analysis. Anesthesiology 2020;133:78–95. 32243326 10.1097/ALN.0000000000003257

[5] Darvall JN , Greentree K , Braat MS , Story DA , Lim WK . Contributors to frailty in critical illness: multi‐dimensional analysis of the clinical frailty scale. J Crit Care 2019;52:193–199. 31096100 10.1016/j.jcrc.2019.04.032

[6] Fried LP , Tangen CM , Walston J , Newman AB , Hirsch C , Gottdiener J , et al. Frailty in older adults: evidence for a phenotype. J Gerontol A Biol Sci Med Sci 2001;56:M146–M156. 11253156 10.1093/gerona/56.3.m146

[7] Rockwood K , Mitnitski A . Frailty in relation to the accumulation of deficits. J Gerontol A Biol Sci Med Sci 2007;62:722–727. 17634318 10.1093/gerona/62.7.722

[8] Rockwood K , Song X , MacKnight C , Bergman H , Hogan DB , McDowell I , et al. A global clinical measure of fitness and frailty in elderly people. CMAJ 2005;173:489–495. 16129869 10.1503/cmaj.050051 PMC1188185

[9] Theou O , Brothers TD , Mitnitski A , Rockwood K . Operationalization of frailty using eight commonly used scales and comparison of their ability to predict all‐cause mortality. J Am Geriatr Soc 2013;61:1537–1551. 24028357 10.1111/jgs.12420

[10] Blodgett J , Theou O , Kirkland S , Andreou P , Rockwood K . Frailty in NHANES: comparing the frailty index and phenotype. Arch Gerontol Geriatr 2015;60:464–470. 25697060 10.1016/j.archger.2015.01.016

[11] Li X , Ploner A , Wang Y , Magnusson PK , Reynolds C , Finkel D , et al. Longitudinal trajectories, correlations and mortality associations of nine biological ages across 20‐years follow‐up. Elife 2020;9. 10.7554/eLife.51507 PMC7012595 32041686

[12] Kanbay M , Segal M , Afsar B , Kang DH , Rodriguez‐Iturbe B , Johnson RJ . The role of uric acid in the pathogenesis of human cardiovascular disease. Heart 2013;99:759–766. 23343689 10.1136/heartjnl-2012-302535

[13] Grayson PC , Kim SY , LaValley M , Choi HK . Hyperuricemia and incident hypertension: a systematic review and meta‐analysis. Arthritis Care Res 2011;63:102–110. 10.1002/acr.20344 PMC3016454 20824805

[14] Holme I , Aastveit AH , Hammar N , Jungner I , Walldius G . Uric acid and risk of myocardial infarction, stroke and congestive heart failure in 417,734 men and women in the Apolipoprotein MOrtality RISk study (AMORIS). J Intern Med 2009;266:558–570. 19563390 10.1111/j.1365-2796.2009.02133.x

[15] Babio N , Martínez‐González MA , Estruch R , Wärnberg J , Recondo J , Ortega‐Calvo M , et al. Associations between serum uric acid concentrations and metabolic syndrome and its components in the PREDIMED study. Nutr Metab Cardiovasc Dis 2015;25:173–180. 25511785 10.1016/j.numecd.2014.10.006

[16] Sautin YY , Johnson RJ . Uric acid: the oxidant‐antioxidant paradox. Nucleosides Nucleotides Nucleic Acids 2008;27:608–619. 18600514 10.1080/15257770802138558 PMC2895915

[17] Duan X , Ling F . Is uric acid itself a player or a bystander in the pathophysiology of chronic heart failure? Med Hypotheses 2008;70:578–581. 17689199 10.1016/j.mehy.2007.06.018

[18] Waring WS , Webb DJ , Maxwell SR . Systemic uric acid administration increases serum antioxidant capacity in healthy volunteers. J Cardiovasc Pharmacol 2001;38:365–371. 11486241 10.1097/00005344-200109000-00005

[19] Bowman GL , Shannon J , Frei B , Kaye JA , Quinn JF . Uric acid as a CNS antioxidant. J Alzheimers Dis 2010;19:1331–1336. 20061611 10.3233/JAD-2010-1330 PMC2859185

[20] Paganoni S , Zhang M , Quiroz Zárate A , Jaffa M , Yu H , Cudkowicz ME , et al. Uric acid levels predict survival in men with amyotrophic lateral sclerosis. J Neurol 2012;259:1923–1928. 22323210 10.1007/s00415-012-6440-7 PMC4441749

[21] Kweon S , Kim Y , Jang MJ , Kim Y , Kim K , Choi S , et al. Data resource profile: the Korea National Health and Nutrition Examination Survey (KNHANES). Int J Epidemiol 2014;43:69–77. 24585853 10.1093/ije/dyt228 PMC3937975

[22] Searle SD , Mitnitski A , Gahbauer EA , Gill TM , Rockwood K . A standard procedure for creating a frailty index. BMC Geriatr 2008;8:24. 18826625 10.1186/1471-2318-8-24 PMC2573877

[23] Kang MG , Kim OS , Hoogendijk EO , Jung HW . Trends in frailty prevalence among older adults in Korea: a nationwide study from 2008 to 2020. J Korean Med Sci 2023;38:e157. 37489714 10.3346/jkms.2023.38.e157 PMC10366411

[24] Kang MG , Jung HW . Association between oral health and frailty in older Korean population: a cross‐sectional study. Clin Interv Aging 2022;17:1863–1872. 36575660 10.2147/CIA.S384417 PMC9790170

[25] Won CW , Lee Y , Lee S , Kim M . Development of Korean Frailty Index for Primary Care (KFI‐PC) and its criterion validity. Ann Geriatr Med Res 2020;24:125–138. 32743333 10.4235/agmr.20.0021 PMC7370789

[26] Kim DH , Glynn RJ , Avorn J , Lipsitz LA , Rockwood K , Pawar A , et al. Validation of a claims‐based frailty index against physical performance and adverse health outcomes in the health and retirement study. J Gerontol A Biol Sci Med Sci 2019;74:1271–1276. 30165612 10.1093/gerona/gly197 PMC6625579

[27] Kim SJ , Jo Y , Park SJ , Ji E , Lee JY , Choi E , et al. Metabolomic profiles of ovariectomized mice and their associations with body composition and frailty‐related parameters in postmenopausal women. J Endocrinol Invest 2024. 10.1007/s40618-024-02338-x 38493245

[28] García‐Esquinas E , Guallar‐Castillón P , Carnicero JA , Buño A , García‐García FJ , Rodríguez‐Mañas L , et al. Serum uric acid concentrations and risk of frailty in older adults. Exp Gerontol 2016;82:160–165. 27394701 10.1016/j.exger.2016.07.002

[29] Sánchez‐Lozada LG , Lanaspa MA , Cristóbal‐García M , García‐Arroyo F , Soto V , Cruz‐Robles D , et al. Uric acid‐induced endothelial dysfunction is associated with mitochondrial alterations and decreased intracellular ATP concentrations. Nephron Exp Nephrol 2012;121:e71–e78. 23235493 10.1159/000345509 PMC3656428

[30] Zhu Y , Hu Y , Huang T , Zhang Y , Li Z , Luo C , et al. High uric acid directly inhibits insulin signalling and induces insulin resistance. Biochem Biophys Res Commun 2014;447:707–714. 24769205 10.1016/j.bbrc.2014.04.080

[31] Li L , Yang C , Zhao Y , Zeng X , Liu F , Fu P . Is hyperuricemia an independent risk factor for new‐onset chronic kidney disease?: a systematic review and meta‐analysis based on observational cohort studies. BMC Nephrol 2014;15:122. 25064611 10.1186/1471-2369-15-122 PMC4132278

[32] Dalrymple LS , Katz R , Rifkin DE , Siscovick D , Newman AB , Fried LF , et al. Kidney function and prevalent and incident frailty. Clin J Am Soc Nephrol 2013;8:2091–2099. 24178972 10.2215/CJN.02870313 PMC3848393

[33] Johnson RJ , Kang DH , Feig D , Kivlighn S , Kanellis J , Watanabe S , et al. Is there a pathogenetic role for uric acid in hypertension and cardiovascular and renal disease? Hypertension 2003;41:1183–1190. 12707287 10.1161/01.HYP.0000069700.62727.C5

[34] Suliman ME , Johnson RJ , García‐López E , Qureshi AR , Molinaei H , Carrero JJ , et al. J‐shaped mortality relationship for uric acid in CKD. Am J Kidney Dis 2006;48:761–771. 17059995 10.1053/j.ajkd.2006.08.019

[35] Hayden MR , Tyagi SC . Uric acid: A new look at an old risk marker for cardiovascular disease, metabolic syndrome, and type 2 diabetes mellitus: the urate redox shuttle. Nutr Metab (Lond) 2004;1:10. 15507132 10.1186/1743-7075-1-10 PMC529248

[36] Beveridge LA , Ramage L , McMurdo ME , George J , Witham MD . Allopurinol use is associated with greater functional gains in older rehabilitation patients. Age Ageing 2013;42:400–404. 23542724 10.1093/ageing/aft046

[37] Taheraghdam AA , Sharifipour E , Pashapour A , Namdar S , Hatami A , Houshmandzad S , et al. Allopurinol as a preventive contrivance after acute ischemic stroke in patients with a high level of serum uric acid: a randomized, controlled trial. Med Princ Pract 2014;23:134–139. 24296871 10.1159/000355621 PMC5586842

[38] Zhou Z , Ryan J , Nelson MR , Woods RL , Orchard SG , Zhu C , et al. The association of allopurinol with persistent physical disability and frailty in a large community based older cohort. J Am Geriatr Soc 2023;71:2798–2809. 37158186 10.1111/jgs.18395 PMC10524392

[39] Ryu KA , Kang HH , Kim SY , Yoo MK , Kim JS , Lee CH , et al. Comparison of nutrient intake and diet quality between hyperuricemia subjects and controls in Korea. Clin Nutr Res 2014;3:56–63. 24527421 10.7762/cnr.2014.3.1.56 PMC3921296

[40] von Haehling S , Coats AJS , Anker SD . Ethical guidelines for publishing in the Journal of Cachexia, Sarcopenia and Muscle: update 2023. J Cachexia Sarcopenia Muscle 2023;14:2981–2983. 38148513 10.1002/jcsm.13420 PMC10751405

