# Supplementary material for: Higher serum uric acid as a risk factor for frailty in older adults: A nationwide population‐based study
Source: J Cachexia Sarcopenia Muscle. 2024 Aug 18;15(5):2134–42. doi: 10.1002/jcsm.13561 (PMC11446678; doi:10.1002/jcsm.13561)
Supplement: Supplementary file 1 — Table S1. Variables included in the frailty index [file JCSM-15-2134-s001.docx]

**Supplementary Table 1.** Variables included in the frailty index

|  | Items |
| --- | --- |
| Comorbidities | 1. Anemia 2. Arthritis 3. Asthma 4. Cancer 5. Cardiovascular disease (myocardial infarction, angina) 6. Cataract 7. Chronic obstructive pulmonary disease 8. Depression 9. Diabetes 10. Dyslipidemia 11. Hypertension 12. Stroke |
| Functional abilities | 1. Activities of daily living limitation 2. Inactivity 3. Difficulty in exercise 4. Difficulty in self-care 5. Difficulty in social activity 6. Hearing impairment 7. Chewing difficulty |
| Signs and symptoms | 1. Anxiety 2. Pain or discomfort 3. Stress 4. Suicidal ideation 5. Weight loss (weight loss of 3 kg or more in the prior year) |
| Laboratory values | 1. Systolic blood pressure (>160 mmHg or <90 mmHg) 2. Diastolic blood pressure (>90 mmHg or <50 mmHg) 3. Hemoglobin (>18 g/dL or <11 g/dL) 4. Blood urea nitrogen (>20 mg/dL or <7 mg/dL) 5. Creatinine (>1.2 mg/dL or <0.6 mg/dL) 6. Fasting glucose (>250 mg/dL or <90 mg/dL) 7. Fasting cholesterol (total cholesterol > 270 mg/dL or < 135 mg/dL) 8. High-density lipoprotein cholesterol (<40 mg/dL) 9. Triglyceride (>200 mg/dL) 10. Proteinuria (urine dipstick test positive) 11. Heart rate irregularity 12. Pulmonary function test abnormality |
| Additional items | 1. Body mass index (<18.5 kg/m^2^ or >25 kg/m^2^) 2. Current smoking |
