# Supplementary material for: Higher serum uric acid as a risk factor for frailty in older adults: A nationwide population‐based study
Source: J Cachexia Sarcopenia Muscle. 2024 Aug 18;15(5):2134–42. doi: 10.1002/jcsm.13561 (PMC11446678; doi:10.1002/jcsm.13561)
Supplement: Supplementary file 2 — Figure S1. Differences in frailty index according to serum uric acid status. A) unadjusted (Men), B) multivariable (age, income, level of education, smoking, hypertension, diabetes, dyslipidemia, stroke, cardiovascular diseases, and body mass index) adjusted (Men). C) unadjusted (Women), D) multivariable (age, income, level of education, smoking, hypertension, diabetes, dyslipidemia, stroke, cardiovascular diseases, and body mass index) adjusted (Women). The estimated means with 95% confidence intervals were generated and compared using general linear model analysis in a complex sample analysis method. Asterisk indicates statistically significant difference from the reference level. Reference level (Men) = serum uric acid ≤ 7.0 mg/dL, Hyperuricemia (Men) = serum uric acid > 7.0 mg/dL, Reference level (Women) = serum uric acid ≤ 6.0 mg/dL, Hyperuricemia (Women) = serum uric acid > 6.0 mg/dL. [file JCSM-15-2134-s003.docx]

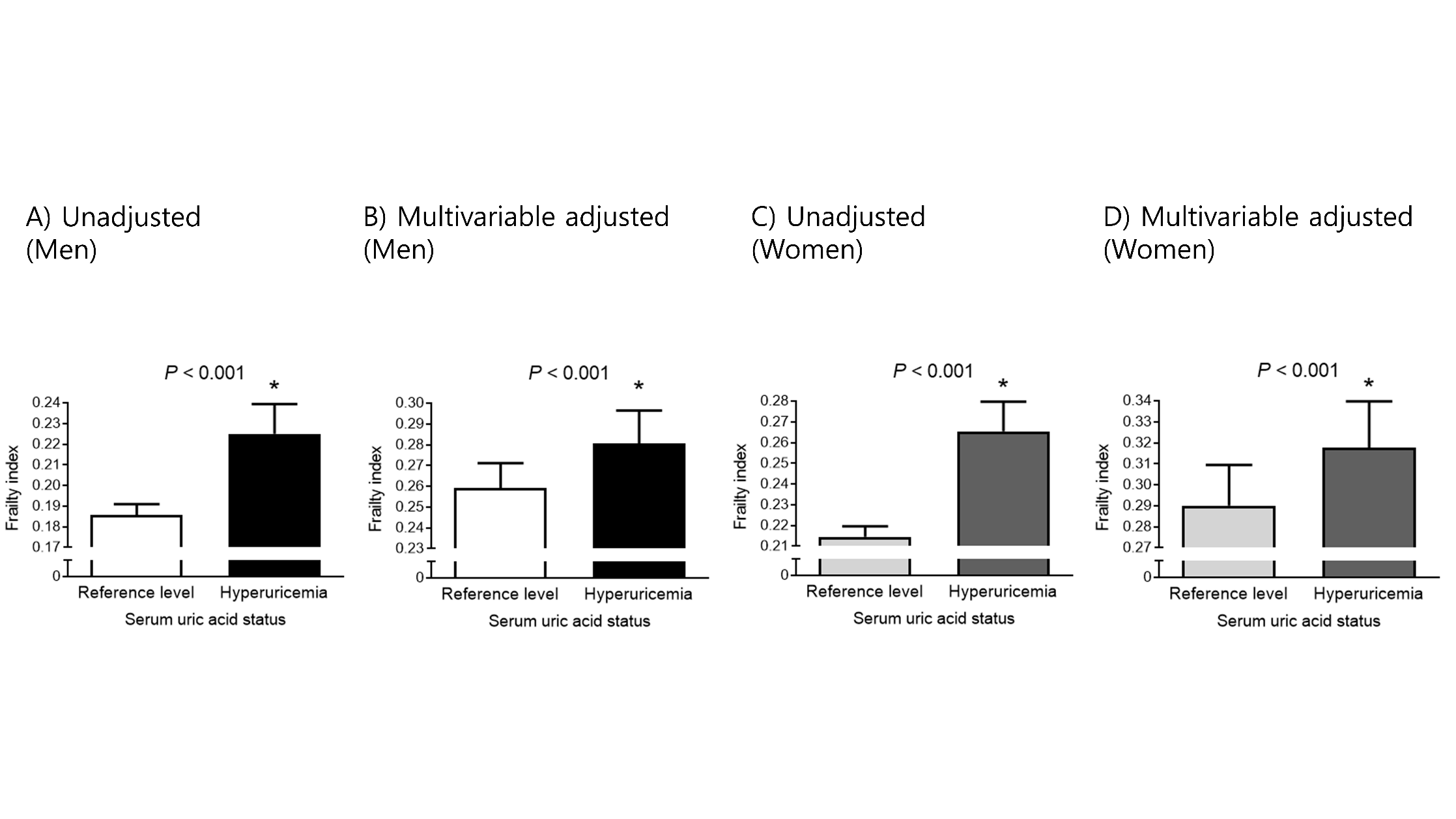


**Supplementary Figure 1.** Differences in frailty index according to serum uric acid status. A) unadjusted (Men), B) multivariable (age, income, level of education, smoking, hypertension, diabetes, dyslipidemia, stroke, cardiovascular diseases, and body mass index) adjusted (Men). C) unadjusted (Women), D) multivariable (age, income, level of education, smoking, hypertension, diabetes, dyslipidemia, stroke, cardiovascular diseases, and body mass index) adjusted (Women). The estimated means with 95% confidence intervals were generated and compared using general linear model analysis in a complex sample analysis method. Asterisk indicates statistically significant difference from the reference level. Reference level (Men) = serum uric acid ≤ 7.0 mg/dL, Hyperuricemia (Men) = serum uric acid > 7.0 mg/dL, Reference level (Women) = serum uric acid ≤ 6.0 mg/dL, Hyperuricemia (Women) = serum uric acid > 6.0 mg/dL.
