# Supplementary material for: Higher serum uric acid as a risk factor for frailty in older adults: A nationwide population‐based study
Source: J Cachexia Sarcopenia Muscle. 2024 Aug 18;15(5):2134–42. doi: 10.1002/jcsm.13561 (PMC11446678; doi:10.1002/jcsm.13561)
Supplement: Supplementary file 3 — Figure S2. Logistic regression analyses to determine the odds ratios for pre‐frail and frail status according to serum uric acid levels. A) unadjusted (Men), B) multivariable (age, income, level of education, smoking, hypertension, diabetes, dyslipidemia, stroke, cardiovascular diseases, and body mass index) adjusted (Men). C) unadjusted (Women), D) multivariable (age, income, level of education, smoking, hypertension, diabetes, dyslipidemia, stroke, cardiovascular diseases, and body mass index) adjusted (Women). OR, odds ratio; CI, confidence interval. Reference level (Men) = serum uric acid ≤ 7.0 mg/dL, Hyperuricemia (Men) = serum uric acid > 7.0 mg/dL, Reference level (Women) = serum uric acid ≤ 6.0 mg/dL, Hyperuricemia (Women) = serum uric acid > 6.0 mg/dL. [file JCSM-15-2134-s002.docx]

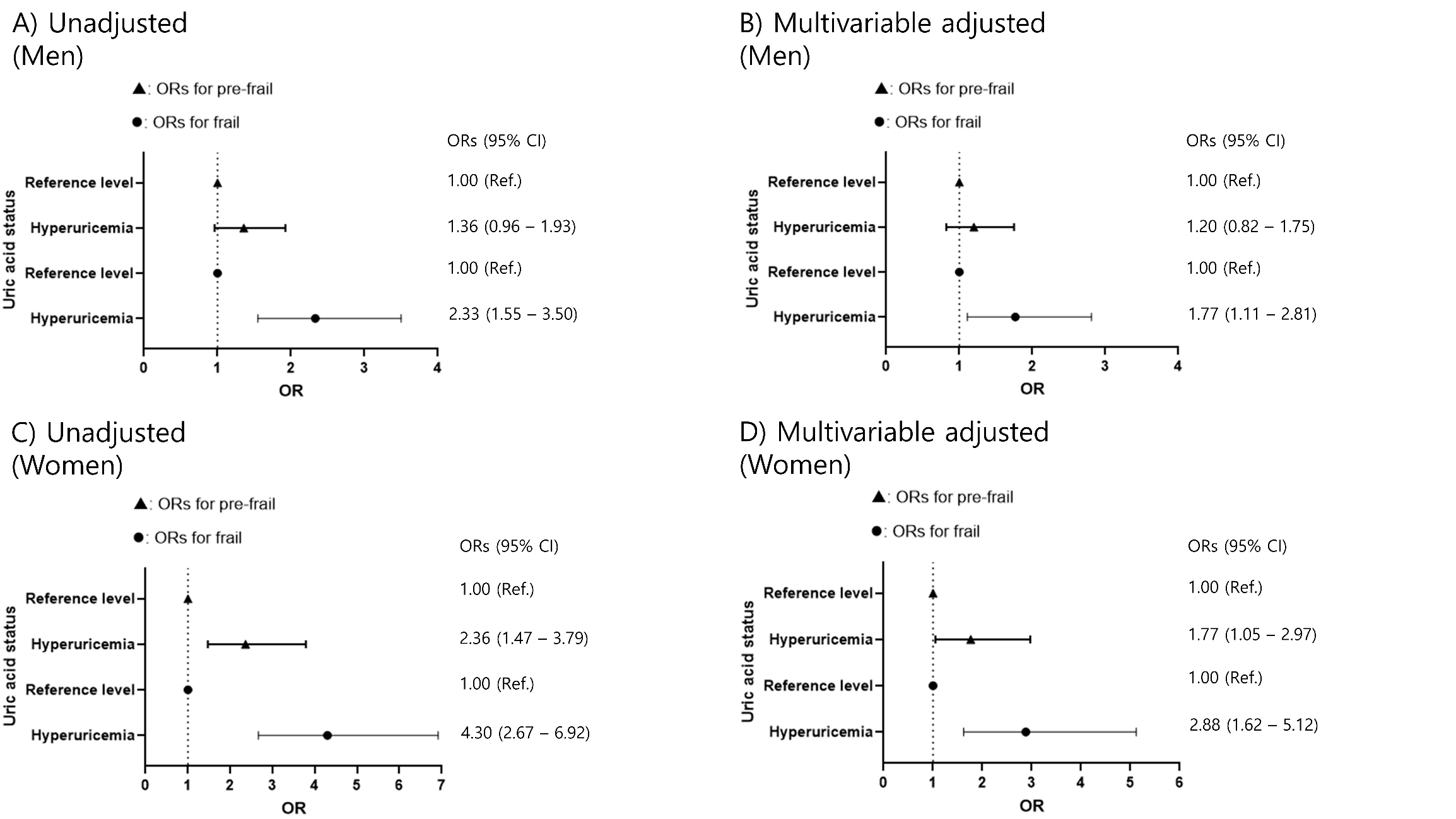


**Supplementary Figure 2.** Logistic regression analyses to determine the odds ratios for pre-frail and frail status according to serum uric acid levels. A) unadjusted (Men), B) multivariable (age, income, level of education, smoking, hypertension, diabetes, dyslipidemia, stroke, cardiovascular diseases, and body mass index) adjusted (Men). C) unadjusted (Women), D) multivariable (age, income, level of education, smoking, hypertension, diabetes, dyslipidemia, stroke, cardiovascular diseases, and body mass index) adjusted (Women). OR, odds ratio; CI, confidence interval. Reference level (Men) = serum uric acid ≤ 7.0 mg/dL, Hyperuricemia (Men) = serum uric acid > 7.0 mg/dL, Reference level (Women) = serum uric acid ≤ 6.0 mg/dL, Hyperuricemia (Women) = serum uric acid > 6.0 mg/dL.
